# Bovine Pericardium Graft as a Salvage Option in Septoplasties at Risk of Septal Perforation

**DOI:** 10.3390/jcm14134592

**Published:** 2025-06-28

**Authors:** Alvaro Sánchez Barrueco, Pilar Benavent Marín, Gonzalo Díaz Tapia, Ignacio Alcalá Rueda, William Aragonés Sanzen-Baker, Luz López Flórez, Jessica Mireya Santillán Coello, José Miguel Villacampa Aubá

**Affiliations:** 1 Rhinology Unit, ENT and Cervicofacial Surgery Department, Hospital Universitario Fundación Jiménez Díaz, 28040 Madrid, Spain; benaventmarinp@gmail.com (P.B.M.); gdiazt@quironsalud.es (G.D.T.); ignacio.alcala@quironsalud.es (I.A.R.); william.aragones@quironsalud.es (W.A.S.-B.); luzlf@quironsalud.es (L.L.F.); jessica.santillan@quironsalud.es (J.M.S.C.); jmvillacampa@fjd.es (J.M.V.A.); 2 Rhinology Unit, ENT and Cervicofacial Surgery Department, Hospital Universitario General de Villalba, 28400 Madrid, Spain; 3 Medicine Faculty, Universidad Alfonso X el Sabio, Villanueva de la Cañada, 28691 Madrid, Spain; 4 Medicine Faculty, Universidad Autónoma de Madrid, 28049 Madrid, Spain

**Keywords:** bovine pericardium, septoplasty, septal perforation, septal deviation, septal heterologous graft

## Abstract

**Background:** Septoplasty is a widely performed surgical procedure to correct nasal septal deviations and improve respiratory function. One of its most significant complications is septal perforation, which can severely impact the patient’s quality of life. This study evaluates the use of bovine pericardium grafts to enhance mucosal healing, thereby reducing the risk of postoperative septal perforation in cases with intraoperative bilateral mucosal defects. **Methods**: A retrospective study was conducted on patients who underwent septoplasty between January 2018 and January 2025 in whom bovine pericardium grafts were interposed due to the presence of bilateral opposing mucosal defects. Epidemiological and surgical variables were recorded, and outcomes and complications were analyzed. **Results**: Out of the 4151 septoplasties performed, 30 cases (0.72%) required bovine pericardium interposition. The mean patient age was 42.87 years. Postoperative absence of septal perforation was confirmed in 90% of cases, with only three postoperative perforations, all asymptomatic and approximately 2 mm in size. Complications were recorded in three patients (10%), all of which were resolved with conservative treatment and without sequelae. **Conclusions**: For the first time in routine surgical practice, bovine pericardium emerges as a viable option for preventing postoperative septal perforation in cases with bilateral opposing mucosal defects. With a high closure rate and a low incidence of adverse events, this material represents a promising tool in septal surgery.

## 1. Introduction

Septoplasty is a widely performed surgical procedure to correct nasal septal deviations and improve nasal respiratory function. It can be performed as a standalone procedure or in combination with other nasal and sinus surgeries, such as septorhinoplasty, functional endoscopic sinus surgery (FESS), or anterior skull base surgery. However, it is not without complications, with septal perforation being one of the most significant. This complication disrupts normal airflow, generating turbulence that can lead to dryness, epistaxis, crusting, and, ultimately, a considerable reduction in quality of life [1].

The incidence of septal perforation following septoplasty ranges between 1% and 6.7% [2,3,4], typically resulting from bilateral mucosal tears during surgery. Additionally, factors such as cocaine or other nasal irritant use and a history of previous septal surgery, infections, or trauma can increase the risk of this complication. Prevention relies on meticulous surgical technique, with careful elevation of the mucoperichondrial and mucoperiosteal flaps, avoiding unnecessary tears, and minimizing the resection of cartilaginous or bony tissue [5].

Once a septal perforation has developed, treatment can be either symptomatic or surgical. The choice of treatment depends primarily on the patient’s symptoms, regardless of perforation size. Asymptomatic perforations are typically managed conservatively with nasal moisturizers, whereas symptomatic perforations often require surgical correction. Various techniques have been used for repair, including sliding flaps and the interposition of fascia and cartilage [6]. More recently, there has been significant progress with endonasal flap techniques [7], particularly mucosal flaps based on the ethmoidal artery [8], the greater palatine artery [9], or combined approaches [10].

The presence of bilateral opposing mucosal dehiscence carries a high risk of postoperative septal perforation, which can increase stress and negatively impact the surgeon’s confidence, potentially worsening surgical outcomes. Therefore, it is essential to have intraoperative tools that can minimize the risk of definitive postoperative perforation. Recent research has explored the use of biomaterials such as bovine pericardium, which has shown promising results in both experimental and clinical studies [11]. Its ability to serve as a scaffold for cellular migration and promote healing suggests that it could be a viable alternative for closing mucosal flap tears, reducing the risk of reperforation and improving surgical outcomes.

Bovine pericardium has previously been used for defect closure in the head and neck [12,13], including anterior skull base surgery, and it has shown positive experimental results in septal applications [11]. Building upon these findings, our study is the first to evaluate the clinical efficacy of bovine pericardium in preventing postoperative septal perforation in patients with intraoperatively identified bilateral opposing mucosal disruptions.

## 2. Materials and Methods

A retrospective analysis was conducted on septal surgeries performed between January 2018 and January 2025 at the Hospital Universitario Fundación Jiménez Díaz and Hospital Universitario General de Villalba. Specifically, data were collected from cases in which the interposition of a heterologous graft between both mucoperichondrial flaps was deemed necessary at the surgeon’s discretion. Inclusion criteria included patients over 18 years of age who underwent septoplasty, either as a standalone procedure or in combination with turbinate and/or sinus surgeries, with a minimum follow-up period of 3 months.

Data potentially influencing surgical outcomes were collected, including epidemiological variables (sex and age) and surgical parameters (type of procedure performed, antibiotic prophylaxis, postoperative antibiotic treatment, complications, and outcome).

Septoplasties were performed using a modified Cottle technique, involving a conservative resection of deviated cartilage and bone, as required. Procedures were carried out under direct visualization with a surgical headlight or microscopic assistance.

**Figure 1:**
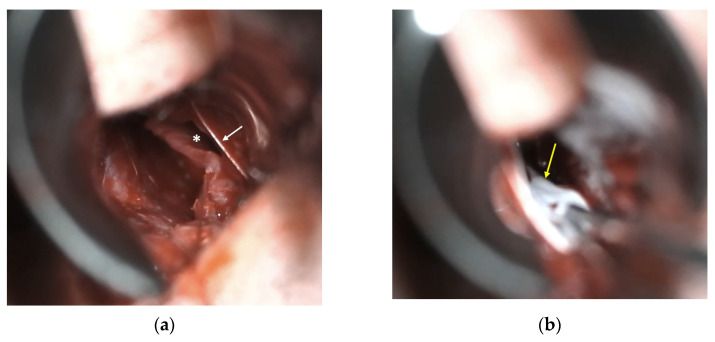
Microscopic intraoperative view: (**a**) left nasal mucosal flap dehiscence (*) and suture of mucosal flap (arrow); (**b**) placement of bovine pericardium graft (arrow) between both nasal mucosal flaps.

If bilateral opposing mucosal defects were observed intraoperatively, a graft was interposed. Bilateral opposing mucosal defects measuring at least 5 mm were considered eligible for graft placement due to their higher likelihood of evolving into septal perforation. The graft was placed between both flaps, completely covering the dehiscence. When possible, the mucosal defect was sutured unilaterally or bilaterally with Polysorb^®^ (Roquette Frères, Lestrem, France) 4-0 to reduce its size (Figure 1a). In all cases, a transfixion suture with Polysorb^®^ 4-0 was performed throughout the nasal septum, including the heterologous graft interposed between the mucosal flaps (Figure 1b). The heterologous graft used in all cases was Tutopatch^®^ (Tutogen Medical GmbH, Neunkirchen, Germany), a biological membrane derived from bovine pericardium, which requires no prior preparation before placement. The complete procedure video can be observed in the Supplementary Materials.

In all cases, paraseptal silicone splints were placed and secured to the nasal septum. Each patient underwent detailed follow-up for at least 3 months, assessed via anterior rhinoscopy and nasofibroscopy.

In some cases, nasal packing was performed using an expandable sponge tampon, which was removed within 24–48 h. In other cases, no nasal packing was performed; instead, cotton pledges soaked in 2% lidocaine with epinephrine were placed and removed 30 min postoperatively. The decision to use nasal packing evolved over time based on the emerging literature discouraging its routine use in septal surgery given its lack of proven benefits in preventing bleeding, hematomas, or residual septal deviation compared to alternative techniques [14].

Statistical analysis was conducted using RStudio (version 4.0.3). Both continuous and categorical variables were analyzed considering data distributions and the nature of the variables involved. A descriptive analysis was performed on the quantitative and categorical variables in the dataset. For categorical variables—such as sex, type of intervention, use of splints, use of nasal packing, prophylactic or postoperative antibiotic use, recorded complications, and outcome—absolute frequencies and relative percentages were calculated.

To analyze the relationship between age (continuous variable) and surgical outcome (categorical variable), the Mann–Whitney U test was applied, as normality assumptions were not met. Fisher’s exact test was applied to assess the relationship between categorical variables, considering the small sample size and low frequencies in certain categories. Additionally, 95% confidence intervals were calculated for each analysis to estimate the strength of associations between categorical variables. A *p*-value < 0.05 was considered statistically significant for all tests.

## 3. Results

During the study period, a total of 4151 septal surgeries were performed at the participating hospitals, excluding cases involving septal perforation repair. These procedures included primary and revision septoplasties and primary and revision septorhinoplasties, with or without unilateral or bilateral synchronous endoscopic nasal and sinus surgery (FESS). Among these, bovine pericardium graft interposition was performed in 30 patients [0.72%]. The cohort consisted of 24 men and six women, with a mean age of 42.87 years (range: 21–68 years).

The most common indication for surgery was primary septal deviation or persistent nasal septal deviation. In total, 80% of cases (n = 24) underwent a primary septoplasty or septorhinoplasty, while the remaining 20% were revision cases.

In all patients, paraseptal silicone splints were sutured to the nasal septum using 3-0 silk sutures. Nasal packing with polyvinyl alcohol sponges was performed in 56.67% of cases (n = 17). Notably, nasal packing was omitted in later cases due to a postoperative protocol change implemented in March 2022 based on prior recommendations [15]. The only exception was cases involving FESS, where the trend towards reducing the use of nasal packing, like septoplasties, has been increasing. In all cases, paraseptal silicone splints were removed between 10 and 21 days postoperatively (median 15 days). The variability in the timing of splint removal is attributable to the retrospective design of the study. However, in the three cases of persistent septal perforation, splint removal occurred between 14 and 17 days postoperatively, similar to the cohort median, suggesting no clear association between splint duration and reperforation.

Prophylactic antibiotics were administered prior to surgery in 50% of cases (n = 15), and postoperative antibiotics were prescribed for 40% of patients (n = 12). Complications were observed in three cases (10%), including one case of fever and rhinorrhea and two cases of significant pain and septal edema. The first case was treated with oral antibiotics (amoxicillin–clavulanate 875/125 mg every 8 h for 7 days) along with oral corticosteroids (prednisone 60 mg every 24 h for 2 days, 20 mg every 24 h for 2 days, followed by 20 mg every 24 h for another 2 days). The remaining two cases had already received postoperative antibiotics and were additionally treated with the same oral corticosteroid regimen. None of these patients developed residual septal perforation.

Across the entire series, three patients (10%) developed a residual septal perforation, which in all cases measured <2 mm and was asymptomatic. The median follow-up duration was 9 months (range: 3–24 months).

None of the analyzed variables, including age (*p* = 0.8102), were significantly associated with postoperative outcomes. The complete dataset is summarized in Table 1.

## 4. Discussion

Septoplasty is a fundamental procedure for nasal surgeons, with septal perforation—alongside surgical site infection—recognized as a potential complication. Intraoperatively, the risk of postoperative perforation can often be anticipated due to difficult tissue dissection, the severity of septal deviation, or, in most cases, the condition of the mucoperichondrial or mucoperiosteal flaps. When bilateral opposing mucosal dehiscence occurs, there are limited options to prevent postoperative perforation beyond direct mucosal closure or an interposition of autologous cartilage. Thus, having a salvage material available for these cases can be highly beneficial.

Septal perforations can significantly impact quality of life [16], which is measurable [17] and can be improved through reconstructive techniques [1]. A perforation impairs nasal function by altering airflow dynamics and reducing the capacity for inspired air humidification [18]. This can lead to symptoms such as nasal dryness, crusting, recurrent epistaxis, and a sensation of obstruction, even in the absence of a true mechanical blockage. The location and size of the perforation play a crucial role, as they influence the magnitude of crossflow air turbulence, causing stress to the surrounding mucosa and exacerbating irritation and nasal discomfort [18].

The use of bovine pericardium in surgery is well-established, with strong clinical outcomes reported in various fields, including cardiac [19], vascular [20], urethral stricture [21], blepharophimosis [22], and cerebrospinal fluid fistula repair [23]. This extensive experience supports its biological plausibility as a graft material in different anatomical areas, including mucosal applications such as the nasal septum.

To date, bovine pericardium has only been used experimentally in the nasal septum, demonstrating stability, minimal antigenicity, and a high capacity for tissue integration without triggering a foreign body inflammatory response [11]. Therefore, our study is the first to evaluate the clinical use of bovine pericardium in preventing postoperative septal perforations in cases where there is an evident risk of residual perforation due to bilateral opposing mucosal dehiscence. With a 90% success rate in 30 cases, bovine pericardium has proven to be a valuable tool for preventing postoperative perforations using a straightforward surgical technique and with a 10% complication rate, none of which resulted in long-term sequelae.

Our study presents several limitations inherent to its design as a retrospective case series. The limited sample size restricts the generalizability of the findings and precludes drawing definitive conclusions. Moreover, the indication for graft placement was based on the surgeon’s intraoperative judgment, which introduces a potential selection bias and variability in case assessment. The absence of a control group without graft placement further limits the strength of the conclusions, although this was considered ethically inappropriate given the high risk of septal perforation in cases with bilateral mucosal defects.

Additionally, the study lacked long-term follow-up and did not include objective, validated patient-reported outcome measures, such as quality-of-life scales, which would have added valuable information regarding the functional impact of the intervention. The lack of comparative data from historical cohorts or other grafting techniques also limits the contextualization of our results.

Future studies should address these limitations by employing prospective, randomized, or multicenter designs with larger sample sizes, standardized selection criteria, and a systematic incorporation of long-term clinical outcomes and patient-reported measures.

## 5. Conclusions

The use of bovine pericardium in septoplasty has proven to be a promising strategy for preventing postoperative septal perforation in cases with bilateral opposing mucosal defects. With a 90% success rate and a low complication profile, it represents a viable and valuable surgical alternative for preserving septal integrity.

## Figures and Tables

**Table 1:** Epidemiological data related to surgical outcomes. ATB (antibiotic).

Variable	% (n)	*p*-Value
Sex *Male* *Female*		
	80% (24)	
	20% (6)	0.8315
Procedure *Primary septoplasty* *Revision septoplasty*		
	80% (24)	
	20% (6)	0.6884
Paraseptal splints *No* *Yes*		
	0% (0)	
	100% (30)	1
Nasal packing *No* *Yes*		
	43.33% (13)	
	56.67% (17)	1
ATB prophylaxis		
*No* *Yes*	50% (15)	
	50% (15)	1
Postoperative ATB		
*No* *Yes*	60% (18)	
	40% (12)	0.7913
Complications		
*No* *Yes*	90% (27)	
	10% (3)	1
Outcome		
*Complete closure* *Perforation < 2 mm*	90% (27)	
	10% (3)	-

## Data Availability

The data that support the findings of this study are available in the file Data.xls, which accompanies this publication.

## Supplementary Materials

The following supporting information can be downloaded at: https://www.mdpi.com/article/10.3390/jcm14134592/s1, Supplementary Video S1. The complete procedure video of the surgical technique and the placement of the bovine pericardium graft under microscopic visualization.

## Abbreviations

The following abbreviation is used in this manuscript:

FESS	Functional Endoscopic Sinus Surgery
